# Highly Sensitive Determination of 2,4,6-Trinitrotoluene and Related Byproducts Using a Diol Functionalized Column for High Performance Liquid Chromatography

**DOI:** 10.1371/journal.pone.0099230

**Published:** 2014-06-06

**Authors:** Burcu Gumuscu, Zeynep Erdogan, Mustafa O. Guler, Turgay Tekinay

**Affiliations:** 1 UNAM, Institute of Materials Science and Nanotechnology, Bilkent University, Ankara, Turkey; 2 MESA+ Institute for Nanotechnology, University of Twente, Enschede, the Netherlands; 3 Life Sciences Application and Research Center, Gazi University, Ankara, Turkey; 4 Department of Medical Biology and Genetics, Faculty of Medicine, Gazi University, Ankara, Turkey; University of Bonn, Institut of experimental hematology and transfusion medicine, Germany

## Abstract

In this work, a new detection method for complete separation of 2,4,6-trinitrotoluene (TNT); 2,4-dinitrotoluene (2,4-DNT); 2,6-dinitrotoluene (2,6-DNT); 2-aminodinitrotoluene (2-ADNT) and 4-aminodinitrotoluene (4-ADNT) molecules in high-performance liquid-chromatography (HPLC) with UV sensor has been developed using diol column. This approach improves on cost, time, and sensitivity over the existing methods, providing a simple and effective alternative. Total analysis time was less than 13 minutes including column re-equilibration between runs, in which water and acetonitrile were used as gradient elution solvents. Under optimized conditions, the minimum resolution between 2,4-DNT and 2,6-DNT peaks was 2.06. The recovery rates for spiked environmental samples were between 95–98%. The detection limits for diol column ranged from 0.78 to 1.17 µg/L for TNT and its byproducts. While the solvent consumption was 26.4 mL/min for two-phase EPA and 30 mL/min for EPA 8330 methods, it was only 8.8 mL/min for diol column. The resolution was improved up to 49% respect to two-phase EPA and EPA 8330 methods. When compared to C-18 and phenyl-3 columns, solvent usage was reduced up to 64% using diol column and resolution was enhanced approximately two-fold. The sensitivity of diol column was afforded by the hydroxyl groups on polyol layer, joining the formation of charge-transfer complexes with nitroaromatic compounds according to acceptor-donor interactions. Having compliance with current requirements, the proposed method demonstrates sensitive and robust separation.

## Introduction

Nitroaromatics are organic contaminants with harmful side effects on a broad range of organisms as underlined by the U.S. Environmental Protection Agency (EPA) in 1989 [1]. 2,4,6-Trinitrotoluene (TNT), one of the most common nitroaromatic compounds, has been used extensively for military purposes after its invention in the late 19^th^ century.

TNT can enter into biological systems and poses a significant risk to human health and other living organisms [2], [3]. It causes induced oxidative stress, resulting in many toxic effects [4], [5], [6]. Contamination level in aquatic systems is established by EPA to a maximum of 2 µg/L [1]. TNT byproducts are of concern since TNT can be transformed to its derivatives such as 2,4-DNT; 2,6-DNT; 2-ADNT; and 4-ADNT, which are also included in military grade products [7]. In last decades, many contaminated sites have been considered to be used as alternative agriculture lands after an extensive decontamination. Therefore, having a cost-effective, high-quality, and sensitive detection method is of utmost importance since “how clean is clean?” question still remains unanswered in remediated areas.

Several analysis methods for identifying minute amounts of TNT have been demonstrated in both biotransformation studies and environmental measurements [8], [9], [10], [11]. Alternative studies such as selective spectrophotometric determination of explosives [12], [13] and nitroaromatic detection via nanoparticles [14] have been also proposed in the past. Among them, chromatographic separation remains as the most preferred technique for quantification of TNT and its byproducts [8]. Being one of the widely used chromatic separation methods, high performance liquid chromatography (HPLC) is a powerful analytical equipment for detection of sub-ppb range concentrations of TNT and TNT derivatives (TATD). The main focus of most of the HPLC studies was to increase sensitivity and reduce separation time and cost [9], [10], [11], [12], [13], [14], [15]. The UV-detector assisted HPLC has been used commonly, as recommended by EPA, because of its predominant advantages such as low toxicity, simplicity, durability, accessibility, rapidity, and cost efficiency [16], [17].

A multitude of chromatography columns has been reported for the separation of nitroaromatics as well as nitramines and nitro esters, including C-18 [18], [19], [20], [21], phenyl [22], and amide columns [10]. However, detection of nitroaromatics by these columns was observed to be challenging because of poor mass-transfer efficiencies and long analysis times. Due to dispersion of metabolites, long waiting periods required prior to separation may mislead calculations of sensitivity. Therefore, use of these columns may fail to satisfy the optimal quantification of nitroaromatics.

In this paper, an HPLC-UV tool was combined with diol column for the first time, inferring further improvement on detection of nitroaromatics. Optimization studies were conducted in order to increase sensitivity and separation capability in notably shortened analysis time. An alternative method to EPA 8330 has been also proposed using C-18 column. Separation performances of diol, phenyl-3 and C-18 columns were compared in terms of standard deviations, detection limits (LOD), quantification limits (LOQ), plate numbers, signal-to-noise ratios, resolutions, and capacity factors. The sensitivity of diol column was evaluated in a field study for validation of the optimized method.

## Experimental Section

### Chemicals and Reagents

The standards of TNT; 2,6-DNT; 4-ADNT; and 2-ADNT (1000 µg/mL in acetonitrile, purity >99.0%) were purchased from SupelCo (USA). An intermediate stock solution of 2,4-DNT was prepared by dissolving powder 2,4-DNT (SupelCo, USA) in acetonitrile with the concentration of 1000 µg/mL. Double distilled water (dH_2_O) (Millipore, USA), HPLC-grade acetonitrile and methanol (Sigma-Aldrich, USA) were used throughout the experiments. Solvents were degassed prior to use. Specimens were stored in glass vials covered with light-blocking plastic bands in order to prevent the degradation by the exposure of sunlight. Stock aliquots and intermediate standard solutions were kept at +4°C in a dark room. Each solution was used for a maximum of 20 days. Calibration solutions of each reagent were prepared by diluting the standard solutions to 1000 µg/L, 500 µg/L, 300 µg/L, 100 µg/L, and 10 µg/L in acetonitrile. Stock solutions were diluted as 1∶10 ratio with acetonitrile. A mix solution was prepared by blending 150 µL of acetonitrile and 10 µL of each standard sample. All samples were prepared fresh on the day of measurement. All stocks were analyzed immediately after preparation.

### Liquid Chromatography Conditions

#### Instrumentation

All measurements were performed on an Agilent 1200 Series HPLC system (Waldbronn, Germany) equipped with 1200 series binary pump (G1310A), micro vacuum degasser (G1322A), standard auto sampler (G13229A), thermostatted column compartment (G1316A), and multi-wavelength detector (G1365D). Ruggedness and reproducibility of the suggested method was evaluated by Accurate Q-TOF LC/MS 6530 (Agilent Technologies, USA) (System properties: Degasser-G1379B, binary pump-G1312B, VWD-G1314B) and TOF LC/MS 6224 (Agilent Technologies, USA) (System properties: Degasser-G1379B, binary pump-G1312A, ASS-G1329A, TCC-G1316A, VWD-G1314B) systems. HPLC analyses were carried out with three different types of columns; Zorbax Eclipse XDB-C18 (4.6 mm×150 mm, 5 µm), Inertsil phenyl-3 (4.6 mm×150 mm, 5 µm) and Inertsil diol (4.6 mm×150 mm, 3 µm). Each column was treated with acetonitrile-water gradient elution solvents. For all measurements, room temperature was used as the system temperature. Bandwidth of the detector was set to 16 nm. Injected sample volume was 5 µL and kept constant for every run. The system was controlled by HP Chemstation software.

#### Proposed chromatographic methods

The reagent peaks were detected by the ultraviolet absorption with a multi wavelength tool at 254 nm [23], [24]. Double distilled water, methanol, and acetonitrile were used as mobile phases. All experiments were replicated at least three times before evaluation. The given values in tables are the averages of experiment sets.

We applied four different methods for the separation of nitroaromatics on the aforementioned columns. In the first method, the mobile phase consisted of a blend of 90% water and 10% acetonitrile. Conditions were the same in all columns. Initial acetonitrile ratio was raised from 10% to 100% in 2–26 min time range. From 26 to 28 min, acetonitrile ratio was gradually reduced to 10% and the total runtime was set to 28 min. In the second method, the same program was employed with methanol instead of acetonitrile in order to evaluate the efficiency of an alternative solvent. The gradient elution method of the third method was described in Table 1 for aforementioned columns. As a fourth separation technique, the procedure shown in Table 1 was employed with the addition of 0.1% formic acid into both mobile phases. After each injection, the system was rinsed by injection of pure acetonitrile by applying method 1. Flow rate of the mobile phases was 0.7 mL/min for all techniques and column types, except for the methods 2 and 3 of phenyl-3 column (flow rate: 1.4 mL/min) and the method 2 of C-18 column (flow rate: 1.2 mL/min). Higher flow rates were used for optimization-oriented methods (2 and 3) of columns with larger particle sizes. The total runtimes listed in Table 1 include re-equilibration time of the detector. Column and technique efficiencies were compared at four levels: 1, evaluation of retention time and separation; 2, assessment of a different mobile phase; 3, effect of formic acid addition to the mobile phase; 4, evaluation of best performances of aforementioned columns.

**Table 1:** Gradient elution methods on diol, C-18, and phenyl-3 columns.

Diol column (Flow rate: 0.7 mL/min)		
Time step (min)	Eluent 1: dH_2_O (90%)	Eluent 2: MeCN (10%)
0	90%	10%
2	90%	10%
18	40%	60%
20	90%	10%
25	90%	10%
**C-18 column (Flow rate: 1.2 mL/min)**		
**Time step (min)**	**Eluent 1: dH_2_O (90%)**	**Eluent 2: MeCN (10%)**
0	50%	50%
5	50%	50%
30	0%	100%
32	50%	50%
35	50%	50%
**Phenyl-3 column (Flow rate: 1.4 mL/min)**		
**Time step (min)**	**Eluent 1: dH_2_O (90%)**	**Eluent 2: MeCN (10%)**
0	80%	20%
2	80%	20%
25	30%	60%
28	80%	20%

### Sample Preparation for Precision Study

Contaminated water and soil samples were collected from a private TNT manufacturing and mine explosion site in Elmadag, Turkey (specific location: +39° 49′ 56.58″, +33° 31′ 5.57″). A special permit was obtained from Mechanical and Chemical Industry Corporation (MKEK) for sample collection from TNT-contaminated lands. The location did not involve endangered or protected species. Spikes for recovery tests were prepared using real samples, originally containing non-zero values of analytes. For recovery tests, samples were spiked with standard solutions of TATD (20 µg/L) for validation of the developed method. Aliquots were mixed with acetonitrile at 1∶1 ratio and swirled overnight in dark for extraction. Extract aliquots (each 20 µL) were filtered through membrane filters (pore size: 0.2 µm) to eliminate large-sized particles and subsequently analyzed by diol column coupled HPLC system. The spiked-concentration calibration-curves of analytes were established by separation of environmental samples spiked at 1000 µg/L, 500 µg/L, 300 µg/L, 100 µg/L, and 10 µg/L. The calibration curves were calculated by the linear regression equation; *y = mx+c*, where *y* is peak area, *x* is the analyte concentration, *m* is the slope and *c* is the intercept. The peak areas, LODs (S/N = 3) and LOQs (S/N = 5) were provided by the ChemStation software.

## Results and Discussion

### Column Properties

Optimization studies were conducted in order to appraise the best responses for quantification. The separation performances of C-18, phenyl-3, and diol columns were evaluated under the same initial conditions and runtime. In order to obtain a high sensitivity, each analyte was infused directly into the system. All compounds were analyzed under acetonitrile–water gradient elution, which showed the best sensitivity results respect to previous reports [9], [11], [17], [18], [19], [20].

Filling material of diol column consists of a dihydroxypropyl-bonded phase on silica surface. The separation performance of this column is based on charge transfer complex formation according to the donor-acceptor interactions between the analytes and diol groups. In this study, diol column was favored as it displayed shortened runtime compared to the other two columns.

Phenyl-3 column is composed of beads that include phenyl groups attached to the short hydrocarbon ends of silica. Phenyl groups on these beads interact with the analyte by π-π interactions as well as hydrophobic interactions. The aromatic rings are π-rich due to their electron donating properties, enabling an enhanced separation of nitroaromatics.

There has been a significant amount of reports on C-18, indicating that the hydrophobic interactions between filling material and nitroaromatics are fundamental for separation performance of this column [24], [25]. Diol and phenyl-3 columns had higher separation performances compared to C-18.

### Column Performances

#### Diol column

Diol column offers the highest resolution of ADNT and DNT isomers. LOD values for diol column were as follows: 0.88 µg/L for TNT; 0.78 µg/L for 2,4-DNT; 1.17 µg/L for 2,6-DNT; 0.84 µg/L for 2-ADNT; and 0.80 µg/L for 4-ADNT. Diol column yielded enhanced recovery rates and LOQ values (Table 2), also the resolution between isomers was high as demonstrated in Table 3. Diol column is adequate for detecting ppb amounts of TATD, showing the highest S/N values among tested columns [26]. It is important to note that; the separation performance of the amide column reported by Gaurav *et al.* [10] is comparable with this work in terms of lower LOD values within the range of 0.17 and 0.93 µg/L for TNT; 2,4-DNT; 2-ADNT; and 4-ADNT analytes. However, the resolution obtained by Gaurav *et al.* was 7% to 50% lower than this study. When compared to two-phase EPA and EPA 8330 methods, solvent consumption was reduced from ∼30 mL/min to 8.8 mL/min. Analyte usage was decreased from 100 µL to 5 µL using diol column. Besides, resolution was improved by 45% and 49% respect to two-phase EPA and EPA 8330 methods [21].

**Table 2:** Comparison of diol column with C-18 and phenyl-3 columns for the analysis of TATD.

(a) Diol column					
Compound	TNT	2,4-DNT	2,6-DNT	2-ADNT	4-ADNT
Retention time (min)	6.39	9.05	9.84	12.13	12.61
Retention time % RSD	0.96	0.24	0.08	0.26	0.04
Peak area % RSD	1.64	1.82	1.42	7.33	0.81
Recovery (%)	105±3	101±2	102±2	101±3	104±4
LOD, S/N = 3 (µg/L)	0.88	0.78	1.17	0.84	0.80
LOQ, S/N = 10 (µg/L)	2.92	2.60	3.90	2.80	2.67
N	7245	9762	9669	34662	137612
S/N	1989	2413	1099.50	2146	2317.20
k’	3.27	4.87	5.40	6.91	7.35
**(b) C-18 column**					
**Compound**	**TNT**	**2-ADNT**	**4-ADNT**	**2,4-DNT**	**2,6-DNT**
Retention time (min)	10.69	13.58	13.94	14.44	14.61
Retention time % RSD	0.75	0.10	0.07	0.36	0.07
Peak area % RSD	16.72	4.71	7.53	2.72	3.14
Recovery (%)	96±5	92±3	96±1	91±2	95±4
LOD, S/N = 3 (µg/L)	2.14	3.05	2.21	3.95	2.01
LOQ, S/N = 10 (µg/L)	7.16	10	7.38	10	6.71
N	25803	47524	42752	71943	53019
S/N	349	245.60	338.70	189.60	372.50
k'	4.09	5.65	5.63	5.83	5.96
**(c) Phenyl-3 column**					
**Compound**	**2-ADNT**	**4-ADNT**	**2,4-DNT**	**2,6-DNT**	**TNT**
Retention time (min)	14.57	15.14	15.75	16.08	17.43
Retention time % RSD	0.51	0.72	0.41	0.09	0.05
Peak area % RSD	1.48	4.62	1.70	2.38	0.41
Recovery (%)	97±3	94±2	98±4	95±3	96±2
LOD, S/N = 3 (µg/L)	0.81	1.32	0.62	1.31	0.83
LOQ, S/N = 10 (µg/L)	2.70	4.42	2.05	4.37	2.77
N	50045	54005	54560	57132	61797
S/N	926.20	565.50	1216.50	570.90	901.80
k’	4.39	4.60	4.83	4.95	5.45

**Table 3:** Selectivity factor and resolution values for diol, C-18, and phenyl-3 columns.

		Selectivity factor (α)	Resolution (R_S)_
Diol column	2,4-DNT and 2,6 DNT	1.11	2.06
	2-ADNT and 4-ADNT	1.06	2.42
C-18 column	2,4-DNT and 2,6 DNT	1.02	1.03
	2-ADNT and 4-ADNT	0.74	1.36
Phenyl-3 column	2,4-DNT and 2,6 DNT	1.03	1.05
	2-ADNT and 4-ADNT	1.23	2.17

Analysis time of diol column was 12% and 40% shorter than C-18 and phenyl-3 columns. Total solvent consumption by phenyl-3, C-18, and diol columns was 33 mL/min, 17.5 mL/min, and 8.8 mL/min, respectively. The results indicate that diol column is the best choice among the tested columns because of its optimal resolution capacity, cost-efficiency and lower toxicity.

#### C-18 column

In this work, an alternative HPLC-UV method for C-18 column has been proposed for detection of nitroaromatics. This method is superior with less sample consumption and inferior with higher detection limits to the method 8330 recommended by the EPA [1]. The solvent consumption was 30 mL/min for EPA 8330 method, 26.4 mL/min for two-phase EPA method while 17.53 mL/min for C-18 column. The sample usage was reduced from 100 µL to 5 µL [21]. In chromatogram of C-18 column, peaks of the reagents displayed minor overlap by the method presented in Table 1. Each reagent eluted in a predicted order under the reversed phase conditions as follows: TNT (10.69 min); 2-ADNT (13.58 min); 4-ADNT (13.93 min); 2,4-DNT (14.43 min); and 2,6-DNT (14.61 min). Resolution values of C-18 column were 1.36 for ADNTs and 0.74 for DNTs. Therefore, the analysis of TATD is not satisfactory for C-18 column with the described method. We observed relatively poor separation of TATD by C-18 column, with low S/N values for TNT (349.00); followed by 2-ADNT (245.60); 4-ADNT (338.70); 2,4-DNT (189.60); and 2,6-DNT (372.50) (Table 2). Even though the selectivity factor of C-18 column was high (1.03), low-resolution performances were noted (Table 3). Using C-18 column, LOD was between 2.01–3.95 µg/L with the optimized method. According to our results, this column turned out to be less convenient for the analysis of TATD.

#### Phenyl-3 column

Phenyl-3 column provided sensitive separation of dinitrotoluene and aminodinitrotoluene isomers (Table 2). Detection limits of phenyl-3 column were between 0.62–1.32 µg/L. The molecule separation by phenyl-3 column was fast, however peaks of DNTs overlapped approximately 2% of their resolution. While S/N values were lower than C-18 column, the selectivity factor for phenyl-3 column was 1.04. High-resolution values suggest that this column can be utilized for quantitative analysis of TATD. In contrast, the overall performance of phenyl-3 column was inferior to diol column by means of an effective analysis and quick handle of separation.

### Separation Mechanisms

The main difference between TNT and its derivatives is rooted in the positioning of nitro groups on the benzene ring. The formation of charge-transfer complex between the column material and analytes is the dominant separation mechanism for DNT isomers, ADNT isomers, and TNT.

**Figure 1:**
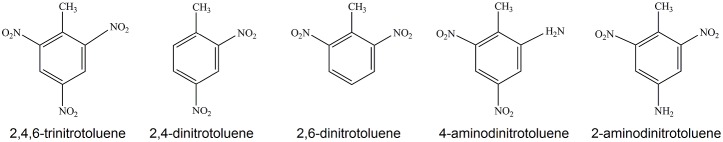
Molecular structures of TNT and its byproducts.

**Figure 2:**
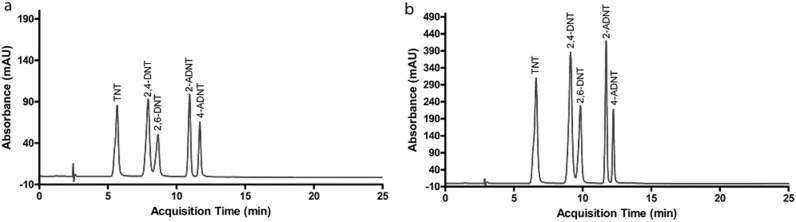
Separation of nitroaromatics by diol column: (a) self-optimization performance, (b) with the method presented in **Table 1**.

The hydroxyl groups in diol column are electron donors. Aromatic amines are electron acceptors, having the ability to undergo charge-transfer complexation with dative and no bond resonance hybrid structures. The nitro group of aromatic amines consists of highly electronegative N and O atoms, which cause the polarization of N−O bond [27]. The nitro group polarization leads the formation of charge-transfer complexes between nitro group of analyte and the column material [28]. Accordingly, the donor-acceptor interactions between the acceptor nitro group and the donor hydroxyl group become the driving force for separation [29], [30]. For TNT, the strong electron affinity of −NO_2_ groups draw the 2, 4, and 6 positions to be electron deficient; therefore, the nitrogen atoms with a lone pair of electrons in amines donate electrons to form n→π charge-transfer complexes [14], [31]. TNT and its byproducts have high charge-transfer capabilities [29]. The order of the relative charge transfer capability for TATD is thought to be TNT; 2,4-DNT; 2-6-DNT; 2-ADNT; and 4-ADNT; as TNT is the highest [32]. Therefore, TNT retarded before than 2,4-DNT. The hydrogen bonds of 2,6-DNT are in ortho position and have effect on the retention of this molecule later than 2,4-DNT. Next, hydrogen bonds in amino groups might have a negative effect in charge transfer capability of ADNT molecules. The amino groups in 4-ADNT are in free position and the molecule is relatively a weaker acceptor, consequently 4-ADNT was observed after 2-ADNT (Fig. 1 and 2).

**Figure 3:**
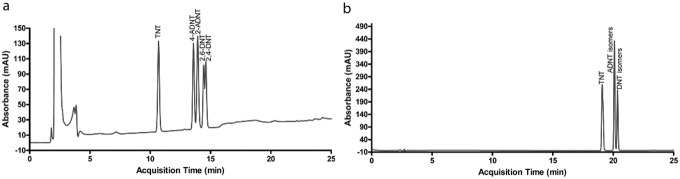
Separation of nitroaromatics by C-18 column: (a) self-optimization performance, (b) with the method presented in **Table 1**.

The separation in C-18 column occurs via hydrophobic interactions. TNT is the most hydrophobic molecule among the tested reagents, thus the TNT peak was observed earlier than the other analytes. The amino group is less polar in 4-ADNT than the nitro group in TNT as a result, 4-ADNT was observed after TNT molecule. 2-ADNT was detected after 4-ADNT because of the methyl group attraction between nitro and amino groups in 2-ADNT. DNTs have two polar groups on the aromatic ring, thus eluted last. Since less prominent steric effects present more powerful hydrophobic interactions, the methyl group bonding on 2,4-DNT with the filling material is stronger than that of the 2,6-DNT isomer, as 2,4-DNT has only one neighboring nitro group (Fig. 3).

**Figure 4:**
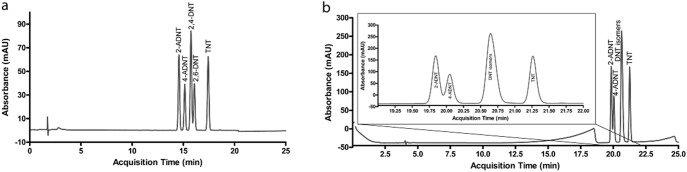
Separation of nitroaromatics by phenyl-3 column: (a) self-optimization performance, (b) with the method presented in **Table 1**.

The reagent peaks obtained from phenyl-3 were completely separate. However, the retention times were higher than that of diol column. The donor acceptor interaction between column’s phenyl groups and ADNTs’ aromatic rings is comparatively weak. ADNTs were eluted first and DNTs were detected afterwards as expected. The first reason for that, the electron-donating groups are not present in the DNTs, resulting in relatively strong adhesion of DNT to the filling material. Secondly, the interaction between the filling material and TNT’s nitro groups is stronger than that of the other analytes because π bonds are not present in TNT. Therefore, TNT was the last molecule to pass through the column (Fig. 4).

### Optimization Studies

Methanol was substituted for acetonitrile as a cheaper alternative in mobile phase optimization. Neither hydrophilic interactions nor hydrogen bonds are formed in between column filling material and the eluent phase (methanol-water), hence shorter retention times were observed for both diol and phenyl-3 columns. Despite its economic advantage and lower toxicity, methanol usage produced more noise and overlapped analyte peaks compared to acetonitrile, which was selected as one of the mobile phases. Gradient elution was applied in all experiments for achieving the best separation, peak shape, and sensitivity in the shortest time possible. Acetonitrile percentages were assigned carefully considering the formation of overlaps and elution of the most retained analytes.

To obtain enhancement in elution over the regular mobile phases, the effect of formic acid at 0.1% (v/v) was evaluated in both mobile phases –water and acetonitrile– by the methods demonstrated in Table 1. Introducing formic acid to the system shortened the retention time as expected while the reagent peaks on diol and phenyl-3 columns overlapped with each other (data not shown). It is important to note that C-18 column presented relatively high separation efficiency. Table 2-b and Fig. 3-a summarize the separation results by C-18 column. While the separation performance of C-18 column increased with the addition of formic acid, it was still lower than diol and phenyl-3 performances, in which no formic acid was utilized. Formic acid protonates the polar amino groups; as a result, more durable interactions are observed between the non-end-capped silica surface and the polar groups, resulting in increased elution capacity of C-18 column.

The flow rates were studied from 0.5 to 1.4 mL/min for all columns and 0.7 mL/min for diol column, 1.4 mL/min for phenyl-3 column and 1.2 mL/min for C-18 column were found to be optimum. The injection volumes were evaluated from 1–10 µL and 5 µL was appeared to be optimum.

### Validation of the Method

#### Calibration curve, accuracy, robustness, sensitivity, and overall performance

For accuracy tests of diol column, the proposed method was applied to the reagents obtained from SupelCo and all samples were spiked at known concentrations (Table S1, Figure S1). Figure S2 shows a linear regression model of different analyte concentrations that were obtained by the proposed procedure. Despite the peaks of ADNT isomers were the closest to each other amongst tested analytes, the selectivity factor of ADNTs was close to 1, ensuring that the analysis of diol column is robust (Table 3). The calibration curve results ensured that the adequate analyte identification was achieved [24], [25], [26], [27], [28], [29], [30], [31], [32], [33]. In addition, the use of a LC/QTOF as well as a LC/TOF system yielded the same results with the HPLC-UV system, excluding trivial concentration and retention time differences due to the conditioning of those systems (data not shown). One of the most sensitive separation methods is reported in this study that allows a precise quantification of analytes [11], [12], [13], [14], [15], [16], [17], [18], [19], [20], [21], [22].

#### Precision study

Real samples, originally containing non-zero levels of TATD, were analyzed by diol column coupled with HPLC system in order to validate the repeatability and intermediate precision. The proposed method was also successfully applied in our previous study on TNT degradation [32]. Repeatability was tested using the same analytes spiked at three different concentrations, evaluating triple injection of five samples on the same day under the same conditions. Intermediate precision was tested using the same process flow for five days. Comparing with commercial analytes, no major changes were observed at retention times of analytes that derived from soil samples. For all TATD, 95–98% recovery was obtained from soil sample extracts. Lower percentage recovery rates can be attributed to electron deficient cationic moieties in water samples or solvent loss in the extraction step [26]. Recoveries of contaminated water are shown to be in between 70–120% in Table 4, assuring the trueness of the methodology.

**Table 4:** Recovery rates and retention times of analytes by diol column.

Analyte	Spiked concentration (µg/L)	Intraday recovery (µg/L) (RSD%)	Interday recovery (µg/L) (RSD%)	Recovery %	Retention time (min)
TNT	20	19.32 (2.4)	19.55 (3.9)	96.60	6.42
2,4-DNT	20	19.42 (3.5)	19.31 (4.1)	97.10	9.09
2,6-DNT	20	19.10 (2.2)	19.12 (2.3)	95.50	9.80
2-ADNT	20	19.66 (3.1)	19.58 (3.7)	98.30	12.16
4-ADNT	20	19.55 (2.2)	19.63 (2.5)	97.75	12.70

## Conclusions

Diol, C-18 and phenyl-3 columns were used in an HPLC-UV system for identification of TNT and TNT derivatives. Diol column showed remarkable enhancement in the subsequent separation of TATD compared to the other columns. The results in terms of analysis time, sensitivity, precision, accuracy, and robustness indicate that diol column is sustainable for monitoring nitroaromatic molecules. The sensitivity of diol column was in the range of 0.78–1.17 µg/L for TATD, showing the highest S/N ratios amongst tested columns and EPA 8330 method. The resolution enhancement for diol column was 45% and 49% in comparison with two-phase EPA and EPA 8330 methods, respectively. Analyte injection volume was reduced to 5 µL. Solvent usage was 30 mL/min for EPA 8330, 26.4 mL/min for two-phase EPA; 17.5 mL/min for C-18, and 33 mL/min for phenyl-3 while it was 8.8 mL/min for diol column. The usage of diol column is highly promising for the separation of nitroaromatics by means of reduction in solvent consumption and non-hazardous chemical waste release. This study offers an efficient and economical alternative to EPA 8330 and two-phase EPA methods in terms of optimal resolution capacity, reduced toxicity and cost-efficiency.

## Supporting Information

Figure S1 Chromatograms obtained from the application of the proposed method to (a) blank sample (ACN) and (b) a standard addition solution spiked at LOD value.
(TIF)

Figure S2 Calibration curves of (a) TNT; (b) 2-ADNT; (c) 4-ADNT; (d) 2,4-DNT; and (e) 2,6-DNT for diol column.
(TIF)

Table S1 Comparison of the nominal and back-calculated concentrations for diol column.
(XLSX)
