# Supplementary figures and images for: Highly Sensitive Determination of 2,4,6-Trinitrotoluene and Related Byproducts Using a Diol Functionalized Column for High Performance Liquid Chromatography
Source: PLoS One. 2014 Jun 6;9(6):e99230. doi: 10.1371/journal.pone.0099230 (PMC4048276; doi:10.1371/journal.pone.0099230)

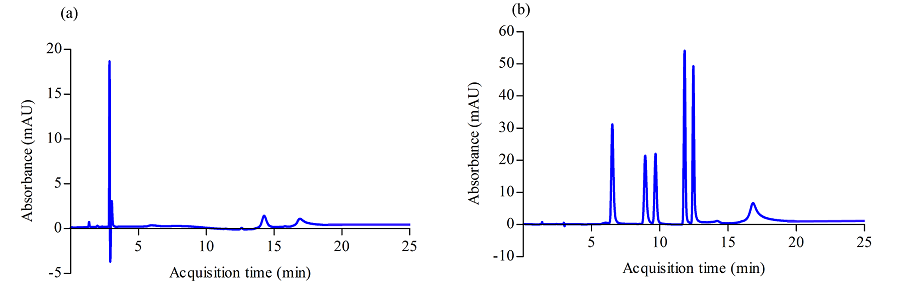

Supplement: Figure S1 — Chromatograms obtained from the application of the proposed method to (a) blank sample (ACN) and (b) a standard addition solution spiked at LOD value. (TIF) [file pone.0099230.s001.tif]

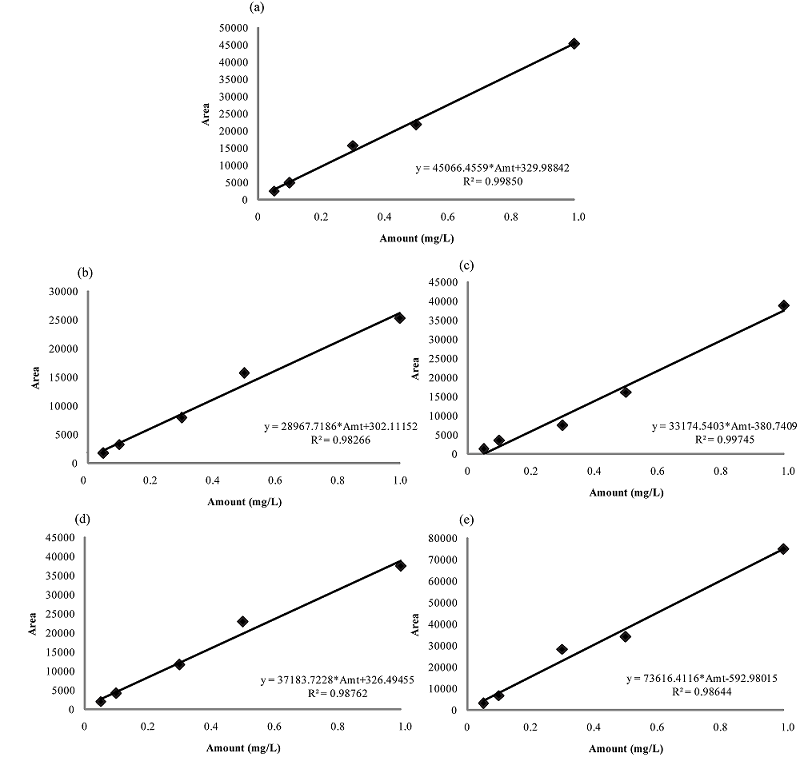

Supplement: Figure S2 — Calibration curves of (a) TNT; (b) 2-ADNT; (c) 4-ADNT; (d) 2,4-DNT; and (e) 2,6-DNT for diol column. (TIF) [file pone.0099230.s002.tif]
